# Acute cigarette smoke or extract exposure rapidly activates TRPA1-mediated calcium influx in primary human airway smooth muscle cells

**DOI:** 10.1038/s41598-021-89051-4

**Published:** 2021-05-05

**Authors:** JinHeng Lin, Michael Taggart, Lee Borthwick, Andrew Fisher, Malcolm Brodlie, M. Flori Sassano, Robert Tarran, Michael A. Gray

**Affiliations:** 1 Biosciences Institute, Faculty of Medical Sciences, Newcastle University, Newcastle upon Tyne, NE2 4HH Tyne and Wear UK; 2 Translational and Clinical Research Institute, Faculty of Medical Sciences, Newcastle University, Newcastle upon Tyne, Tyne and Wear UK; 3 Paediatric Respiratory Medicine, Great North Children’s Hospital, Newcastle Upon Tyne Hospitals NHS Foundation Trust, Newcastle upon Tyne, Tyne and Wear UK; 4 Department of Cell Biology and Physiology, School of Medicine, University of North Carolina at Chapel Hill, Chapel Hill, NC USA

**Keywords:** Cell biology, Physiology

## Abstract

Tobacco smoking is the largest risk factor for developing chronic obstructive pulmonary disease (COPD), and is associated with hyperresponsiveness of airway smooth muscle (ASM). Chronic exposure to cigarette smoke (CS) leads to airway inflammation and remodelling. However, the direct effect of gaseous CS or CS extract (CSE) on human airway smooth muscle cell (hASMC) function remains poorly understood. This study investigated the acute effect of CS/CSE on calcium homeostasis, a key regulator of ASM physiology and pathophysiology. Primary hASMC were isolated from non-smoking donor lungs, and subjected to Ca^2+^ imaging studies. We found that both CS, and CSE, rapidly elevated cytosolic Ca^2+^ in hASMC through stimulation of plasmalemmal Ca^2+^ influx, but excluded store-operated and L-type Ca^2+^ channels as mediators of this effect. Using a specific pharmacological inhibitor, or shRNA-driven knockdown, we established that both CS and CSE stimulated Ca^2+^ influx in hASMC through the neurogenic pain receptor channel, transient receptor potential ankyrin 1 (TRPA1). CS/CSE-dependent, TRPA1-mediated Ca^2+^ influx led to myosin light-chain phosphorylation, a key process regulating ASM contractility. We conclude that TRPA1 is likely an important link between CS/CSE exposure and airway hyperresponsiveness, and speculate that acute CS/CSE-induced Ca^2+^ influx could lead to exacerbated ASM contraction and potentially initiate further chronic pathological effects of tobacco smoke.

## Introduction

Tobacco smoking is the largest risk factor for developing COPD, which is associated with airway hyperresponsiveness (AHR), amongst other lung conditions such as emphysema, chronic bronchitis and infections^1–4^. Airway smooth muscle (ASM) is at the centre of AHR and is more sensitive to contractile stimuli in the diseased state. This may be exacerbated by airway inflammation and remodelling, which are extensively linked to tobacco smoking^5–9^.

Cigarette smoke (CS) is a complex chemical mixture, containing over 4000 identified compounds. The most prominent of these are nicotine, acrolein, N-nitrosamines, various volatile compounds and heavy metals^10^. Experimental analysis of CS utilises one or both of its two distinct phases: the gaseous phase and the particulate phase. The gaseous phase entails the smoke that passes through a glass fibre filter with 0.3 µm pores, while the particulate phase, also known as the tar phase, consists of the relatively larger aerosol particles trapped in these pores^11^. Delivery of CS can be performed by direct aerosolisation into a closed chamber, commonly used for animal smoking studies, but have also been applied in in vitro experiments. Another delivery model is known as CS extract (CSE), produced by bubbling CS into a buffer solution, which can then be applied to in vitro or ex vivo samples, or collected for chemical analysis. The two delivery models are often reported in the literature as though to be synonymous, but certain volatile constituents potentially lost through vigorous bubbling during the production of CSE are retained in the closed chamber in the CS model.

In vitro experiments have shown that chronic exposure of human ASM cells (hASMC) to CSE augments ROS generation, chemokine secretion, collagen deposition, proliferation, migration, apoptosis and inhibits wound-healing, all of which are hallmarks of the inflammatory profile and airway remodelling in CS-related airway diseases^12–18^. However, CSE, or CS, may also have a direct effect on the intrinsic contractility of the ASM^6^. ASM contractility, and hence the tone and calibre of the airway, is tightly regulated by intracellular Ca^2+^ signalling. Chronic CSE exposure enhances agonist-induced Ca^2+^ release and SOCE in hASMC, which correlates with upregulation of several components of Ca^2+^ signalling, including Ca^2+^ permeable channels, potentially leading to long-term disruption of Ca^2+^ homeostasis^16,19^. In relation to ASM contractility, there are varying effects reported of chronic CS or CSE exposure. Chronic in vivo CS exposure sensitises the responsiveness of rat bronchial SM to acetylcholine ex vivo^20^. Interestingly, rat bronchial segments cultured with CSE chronically are found to be hyper-contractile^15,21^, but bovine tracheal strips and hASMC cultured with CSE exhibit attenuated contractile responses^12,14^. This makes it difficult to extrapolate to what may occur in these circumstances in hASMCs.

On the other hand, Yoon, et al.^12^ reported that transient exposure to CSE increased [Ca^2+^]_i_ in hASMC, but the mechanism of action was not explored. Acute CSE treatment also leads to ex vivo constriction of guinea pig bronchi, but rapid relaxation of mice lung slices^22,23^. Otherwise, the direct effects of acute gaseous CS or CSE exposure on hASMC Ca^2+^ signalling, and subsequent downstream effects, remain poorly understood. In the present study, we therefore explored the effects of acute exposure to CS, or CSE, on Ca^2+^ homeostasis in primary hASMC. Importantly, we applied both the aqueous extract model as well as direct exposure to gaseous CS in order to tease out potential differences between the two delivery methods. Using a variety of pharmacological and molecular interventions, we established that both CS and CSE stimulate Ca^2+^ influx in hASMC through the TRPA1 channel, and lead to downstream activation of contractile signalling mechanisms.

## Results

### Gaseous CS and diluted CSE activate Ca^2+^ influx in hASMC

**Figure 1:**
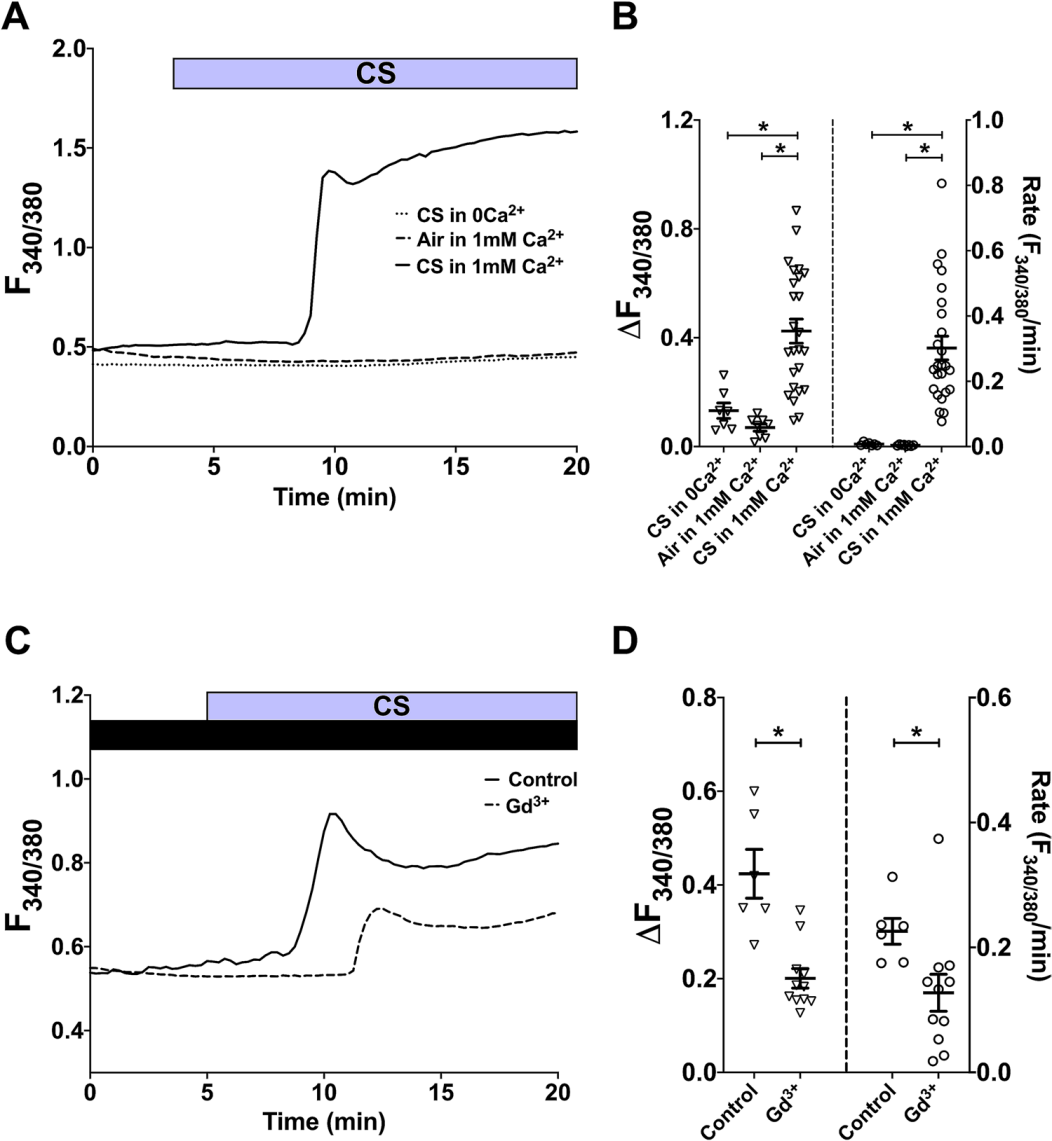
Whole CS activates Ca^2+^ influx in hASMC. (A, C) Representative Ca^2+^ imaging traces from separate experiments tracking changes in [Ca^2+^]_i_ following puffing one whole cigarette with or without extracellular Ca^2+^ **(A)**, or with or without 10-min pre-treatment with 500 µM Gd^3+^, which was also present throughout the experiment **(C)**. **(B,D)** Summary of amplitude and rate of [Ca^2+^]_i_ changes corresponding to experiments in **(A,C)**, respectively, presented as mean ± SEM. One-way ANOVA with Holm-Sidak’s multiple comparisons test was performed amongst the 3 groups (**B**; 4 independent donors; n = 7–25); unpaired t-test was performed between the control group and the Gd^3+^-treated group (**D**; 3 independent donors; n = 6–11). * = p < 0.05. Control in **(C,D)** refers to CS exposure without Gd^3+^ pre-treatment.

To investigate the effect of CS on cytosolic Ca^2+^ concentration in isolated primary hASMC, the cells were exposed to one whole Kentucky 3R4F reference cigarette as described above. Real-time changes in [Ca^2+^]_i_ was reported by changes in fura-2 fluorescence ratio, and the amplitude (peak [Ca^2+^]_i_ minus baseline [Ca^2+^]_i_) and rate (change in [Ca^2+^]_i_ per minute fitted with linear regression) of the calcium response were calculated from each individual experiment. In the presence of 1 mM extracellular Ca^2+^, exposure to the gaseous phase of one whole cigarette led to a rise in [Ca^2+^]_i_ that was significantly higher, in both amplitude and rate, than the change in [Ca^2+^]_i_ elicited by room air puffed with the same volume/frequency (Fig. 1A,B; p < 0.05, air vs. CS in 1 mM Ca^2+^). This Ca^2+^ response typically started to rise after 4–6 puffs, and reached an initial peak 4.5–8 min after the first puff. More importantly, the CS-induced [Ca^2+^]_i_ increase required extracellular Ca^2+^, as the response was abolished when CS was administered in a nominally Ca^2+^-free solution (p < 0.05, CS in 0Ca^2+^ vs. in 1 mM Ca^2+^). This points to Ca^2+^ influx from the extracellular space as the primary source of the CS-induced [Ca^2+^]_i_ elevation, and that intracellular Ca^2+^ stores are not likely to contribute to this response.

To ascertain if the CS-induced rise in [Ca^2+^]_i_ was through a plasma membrane-bound Ca^2+^ channel, we tested the effect of gadolinium (Gd^3+^), a lanthanide ion that non-specifically blocks Ca^2+^-permeable pathways at the PM. Indeed, both the amplitude (52.6%) and rate (43.6%) of the CS-activated Ca^2+^ response were significantly inhibited by Gd^3+^ (Fig. 1C,D; p < 0.05, control vs. Gd^3+^-treated), suggesting that Ca^2+^ influx through PM-bound channels was the main source of this response.

**Figure 2:**
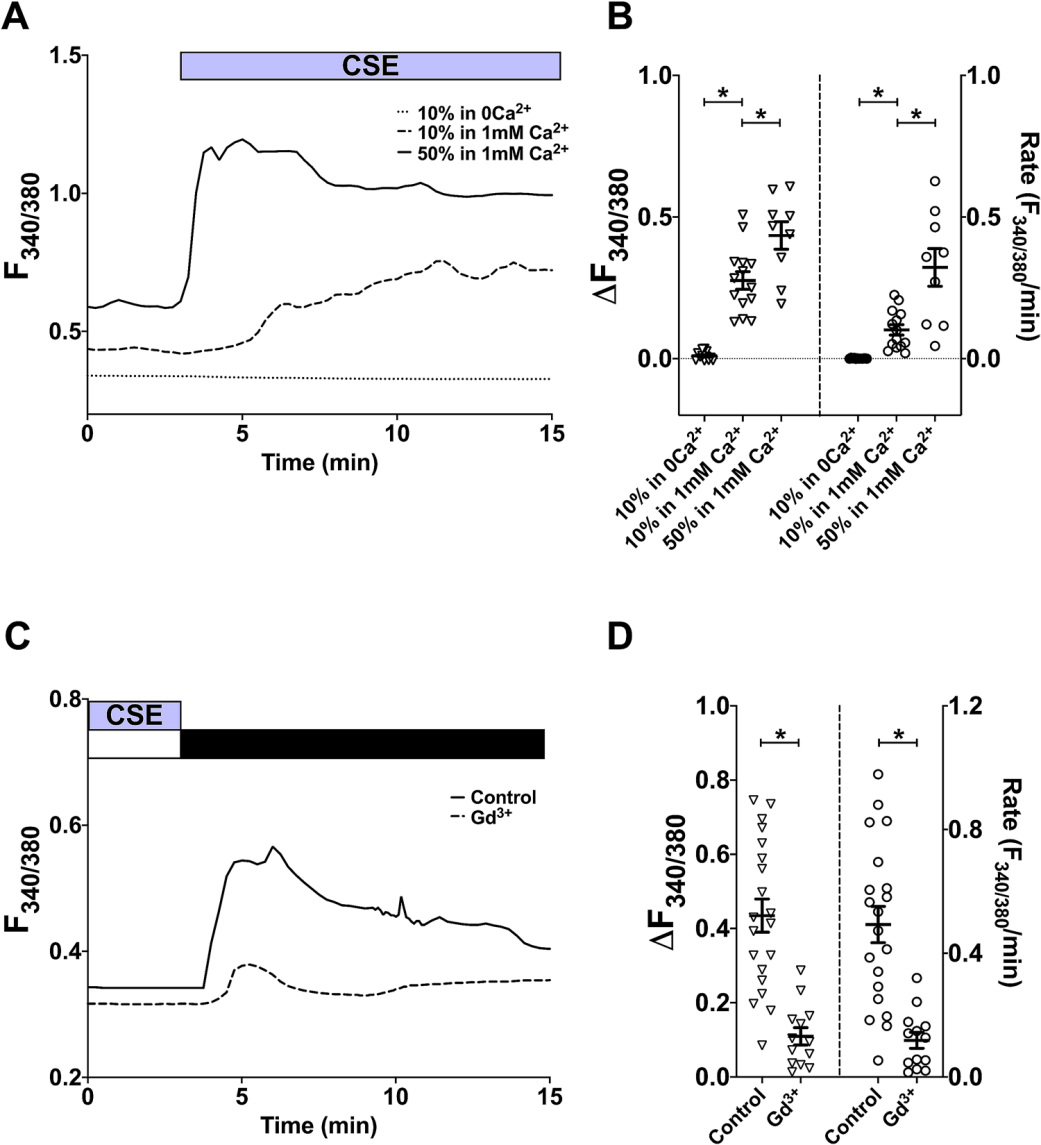
Diluted CSE also activates Ca^2+^ influx in hASMC. **(A,C)** Representative Ca^2+^ imaging traces from separate experiments tracking changes in [Ca^2+^]_i_ following perfusion with diluted CSE with or without extracellular Ca^2+^ **(A)**, or following Ca^2+^ addback (1 mM Ca^2+^; black bar) after 5-min perfusion with 10% CSE in a nominally Ca^2+^-free solution (white bar), with or without 10-min pre-treatment with 100 µM Gd^3+^, which was also present throughout the experiment **(C)**. **(B,D)** Summary of amplitude and rate of [Ca^2+^]_i_ changes corresponding to experiments in **(A,C)**, respectively, presented as mean ± SEM. One-way ANOVA with Holm-Sidak’s multiple comparisons test was ran amongst the 3 groups (**B**; 3 independent donors; n = 9–14); unpaired t-test was performed between the control group and the Gd^3+^-treated group (**D**; 3 independent donors; n = 13–20). *p < 0.05. Control in **(C,D)** refers to CSE-activated Ca^2+^ addback without Gd^3+^ pre-treatment.

To directly compare the effect of CSE to that of gaseous CS, diluted CSE was delivered to hASMC through a perfusion system. Similarly to gaseous CS, exposure to diluted CSE also raised [Ca^2+^]_i_, and this was concentration-dependent (Fig. 2A,B; p < 0.05, 10% vs. 50% CSE in 1 mM Ca^2+^). This response typically reached a peak within 1–2.5 min of exposure to 50% CSE, and 3–6.5 min following exposure to 10% CSE. As for the CS-induced rise in [Ca^2+^]_i_, the CSE-induced change in [Ca^2+^]_i_ was also abolished by the removal of extracellular Ca^2+^ (Fig. 2A,B; p < 0.05, 10% and 50% CSE in 1 mM Ca^2+^ vs. in 0Ca^2+^). Since a 5-min exposure to CSE appeared to be sufficient to activate Ca^2+^ influx, we developed a calcium-addback protocol, whereby cells were first exposed to CSE in 0Ca^2+^ for 5 min to synchronise influx for the whole population of cells, then Ca^2+^ was added back in the absence of CSE. This reduced inter-experiment variability in response profiles.

Experiments with this Ca^2+^ addback protocol revealed that the continual presence of CSE was not required for maintained Ca^2+^ influx, and that the channels remained open following removing of extracellular Ca^2+^ (data not shown). More importantly, following an extended washout period (at least 25 min after removing CSE), both the amplitude and rate of the Ca^2+^ addback response was attenuated but not abolished (data not shown), suggesting that CSE’s activation of the Ca^2+^ influx pathway was likely to be reversible. Using the addback protocol, we examined the effect of Gd^3+^ on CSE-induced Ca^2+^ influx. Gd^3+^ also significantly attenuated both the amplitude (74.8%) and rate (76.0%) of CSE-activated Ca^2+^ influx in hASMC (Fig. 2C,D; p < 0.05 control vs. Gd^3+^-treated), providing further evidence that a PM-bound Ca^2+^ channel was responsible for this response.

Although the general Ca^2+^ response elicited by the two tobacco product delivery methods were similar, the response profiles bore subtle differences. In the presence of 1 mM extracellular Ca^2+^, once cells were exposed to 10% and 50% CSE, [Ca^2+^]_i_ started to increase within a minute; whereas in the CS model, there was a noticeable time lag of 2–3 min (4–6 puffs) before [Ca^2+^]_i_ began to rise. Indeed, on average it took longer for CS-induced Ca^2+^ response to reach a peak than the CSE-induced response (4.5–8 min vs. 1–2.5 min for 50% CSE and 3–6.5 min for 10% CSE).

### CS- and CSE-induced Ca^2+^ influx do not involve store-operated or voltage-gated calcium channels

**Figure 3:**
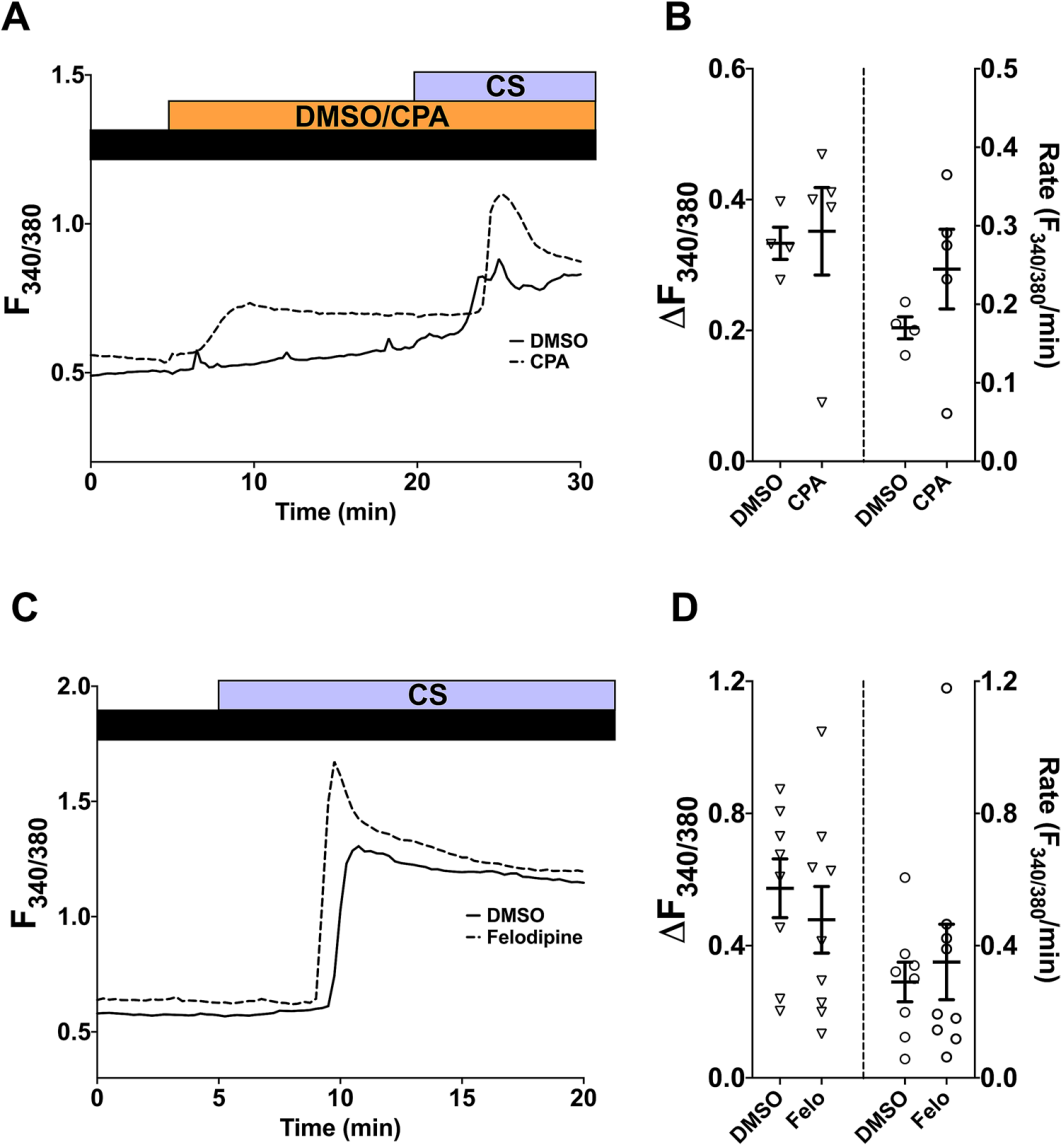
CS-induced Ca^2+^ influx utilises a different pathway from SOCC and LTCC. **(A,C)** Representative Ca^2+^ imaging traces from separate experiments tracking changes in [Ca^2+^]_i_ following puffing one whole cigarette after 15-min treatment with 10 µM CPA **(A)**, or after 10-min pre-treatment with 1 µM felodipine, which was also present throughout the experiment **(C)**. Equal volumes of DMSO (0.01% for CPA, 0.001% for felodipine) were used for vehicle control. Experiments were run in the presence of 1 mM extracellular Ca^2+^ (black bar). **(B,D)** Summary of amplitude and rate of CS-induced [Ca^2+^]_i_ changes corresponding to experiments in **(A,C)**, respectively, presented as mean ± SEM. Unpaired t-test was performed between the CPA-treated group (**B**; 2 independent donors; n = 4–5) or felodipine-treated group (**D**; 3 independent donors; n = 8–9) against the vehicle control group. *p < 0.05.

To explore possible candidates of CS/CSE-activated Ca^2+^ influx pathways in hASMC, we considered the potential contribution of store-operated Ca^2+^ channels (SOCC) and the voltage-gated L-type Ca^2+^ channels (LTCC), both of which play an important role in refilling SR Ca^2+^ stores in ASMC^24^. The involvement of SOCC was studied using CPA (10 µM), a SERCA inhibitor, which depletes SR Ca^2+^ stores and activates SOCC. After store depletion and the subsequent SOC influx reaching a plateau, CS from one cigarette was puffed onto hASMC as previously described. CS was able to instigate an additional elevation in [Ca^2+^]_i_ when SOCC were already active (Fig. 3A), similar to the vehicle control where SOCC were not activated (Fig. 3B; p > 0.05, DMSO vs. CPA-treated).

The potential role of LTCC was examined using felodipine, which specifically blocks LTCC in favour of other voltage-gated Ca^2+^ channels^25^. A 10-min pre-treatment and continual presence of felodipine (1 µM) did not significantly attenuate CS-induced Ca^2+^ influx (Fig. 3C,D; p > 0.05, DMSO vs. felodipine-treated). These results suggest that neither SOCC nor LTCC contribute to the CS-induced Ca^2+^ influx.

**Figure 4:**
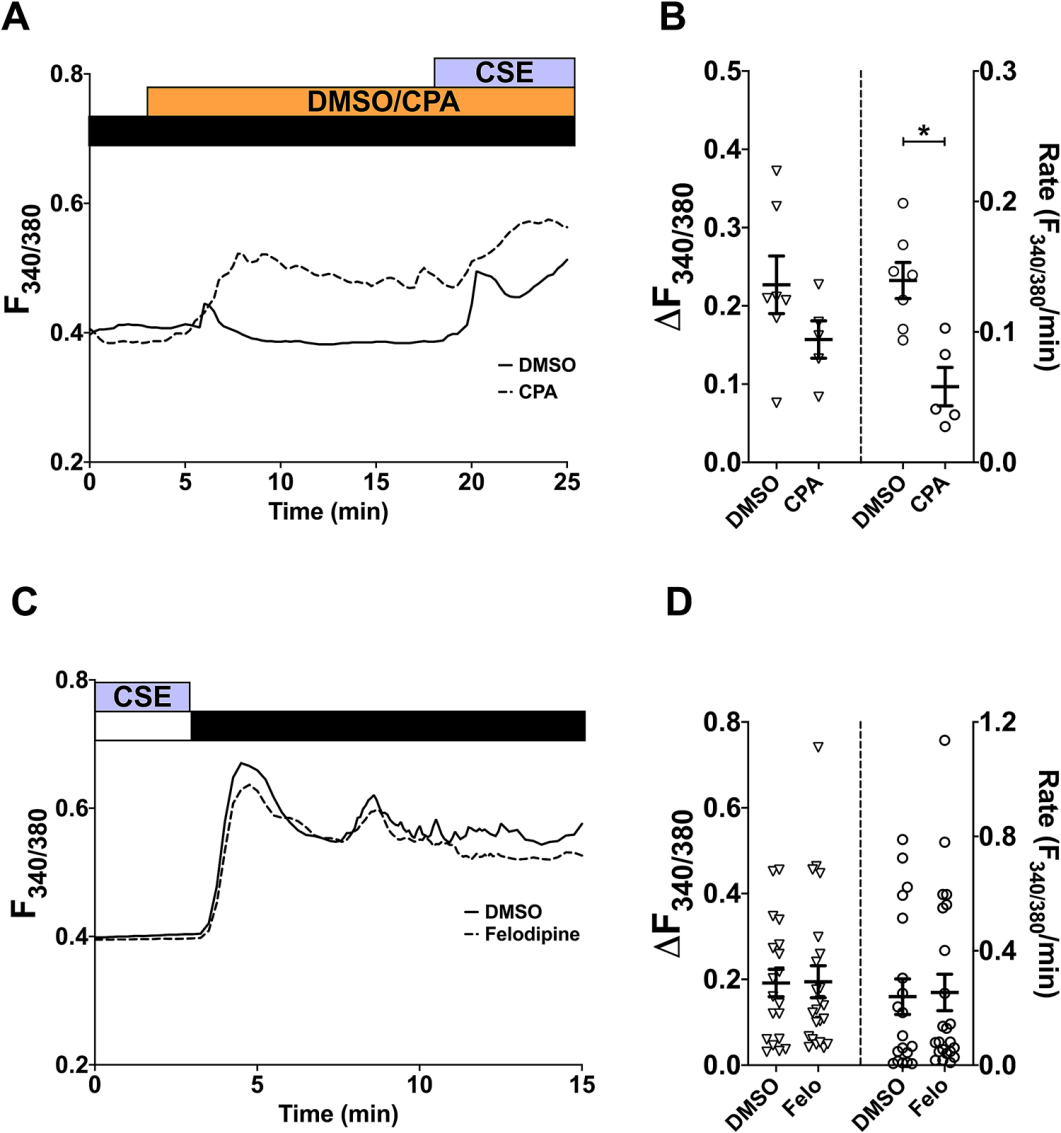
CSE-induced Ca^2+^ influx also utilises a different pathway from SOCC and LTCC. **(A,C)** Representative Ca^2+^ imaging traces from separate experiments tracking changes in [Ca^2+^]_i_ following perfusion with 10% CSE after 15-min treatment with 10 µM CPA **(A)**, or following Ca^2+^ addback (1 mM Ca^2+^; black bar) after 5-min perfusion with 10% CSE in a nominally Ca^2+^-free solution (white bar), with or without 10-min pre-treatment with 1 µM felodipine, which was also present throughout the experiment **(C)**. Equal volumes of DMSO (0.01% for CPA, 0.001% for felodipine) were used for vehicle control. **(B,D)** Summary of amplitude and rate of CSE-induced or Ca^2+^ addback [Ca^2+^]_i_ changes corresponding to experiments in **(A,C)**, respectively, presented as mean ± SEM. Unpaired t-test was performed between the CPA-treated group (**B**; 2 independent donors; n = 5–7) or felodipine-treated group (**D**; 4 independent donors; n = 19–23) against the vehicle control group. *p < 0.05.

The involvement of SOCC and LTCC were also studied using the CSE model. After activating SOCC with CPA, exposure to 10% CSE also led to a further Ca^2+^ response (Fig. 4A). The amplitude of this response after SOCC activation was similar to the vehicle control, but the rate was significantly attenuated after SOCC activation (Fig. 4B; p < 0.05, DMSO vs. CPA-treated).

The role of LTCC was also investigated by including felodipine in the CSE-activated Ca^2+^-addback protocol described above (Fig. 4C). Similar to results in Fig. 3D, neither the amplitude nor the rate of CSE-activated Ca^2+^ influx was significantly attenuated by pre-treatment and continual presence of 1 µM felodipine (Fig. 4D; p > 0.05, DMSO vs. felodipine-treated). Altogether, these data suggest that SOCC and LTCC, two prominent Ca^2+^ channels regulating SMC store refilling, do not make major contributions to the CS/CSE-induced Ca^2+^ influx.

### CS- and CSE-induced Ca^2+^ influx is mediated by the TRPA1 channel

**Figure 5:**
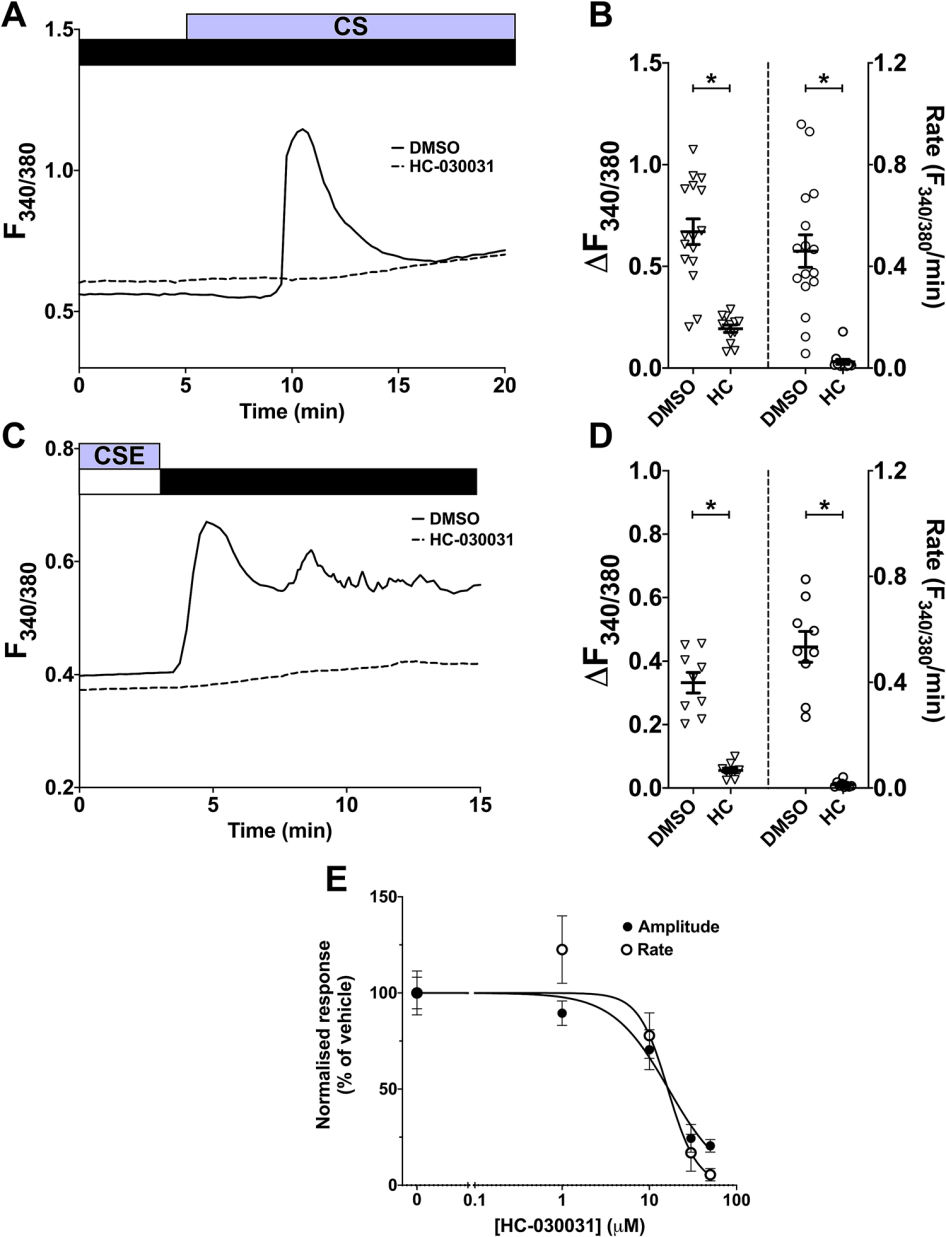
CS and CSE-induced Ca^2+^ influx is inhibited by the TRPA1 channel blocker HC-030031 in a concentration-dependent manner. **(A,C)** Representative Ca^2+^ imaging traces from separate experiments tracking changes in [Ca^2+^]_i_ following puffing one whole cigarette **(A)** or following Ca^2+^ addback after 5-min perfusion with 10% CSE **(C)** with or without 10-min pre-treatment with 50 µM HC-030031, which was also present throughout the experiment. Equal volumes of DMSO (0.1%) were used for vehicle control. **(B,D)** Summary of amplitude and rate of the [Ca^2+^]_i_ increase induced by CS **(B)** or CSE-activated Ca^2+^ addback **(D)**, respectively, presented as mean ± SEM. Unpaired t-test was performed between the vehicle control group and the HC-030031-treated group (3 independent donors; n = 9–16). * p < 0.05. **(E)** Dose–response curves for the normalised amplitude and rate of CS-induced Ca^2+^ influx (normalised to % of the mean amplitude/rate of DMSO control) at different concentrations of HC-030031, presented as mean ± SEM.

After establishing SOCC and LTCC as unlikely targets activated by CS/CSE, a possible alternative candidate was TRPA1, a Ca^2+^-permeable cation channel that has been shown to mediate CSE-induced Ca^2+^ increases in other cell types^23,26–28^. We used the inhibitor HC-030031 to study the involvement of TRPA1. Pre-treatment with HC-030031 (50 µM) significantly inhibited the amplitude and rate of both CS- (71.2/94.7% inhibition) and CSE- (69.0/94.0% inhibition) induced Ca^2+^ influx in hASMC (Fig. 5A–D; p < 0.05, DMSO vs. HC-030031). This inhibition was concentration-dependent, and the IC_50_ for blocking CS-induced Ca^2+^ influx was 16.6 µM for amplitude and 16.3 µM for rate (Fig. 5E). Such significant inhibition by a potent TRPA1 inhibitor suggests TRPA1 is a major facilitator of CS/CSE-activated Ca^2+^ influx.

**Figure 6:**
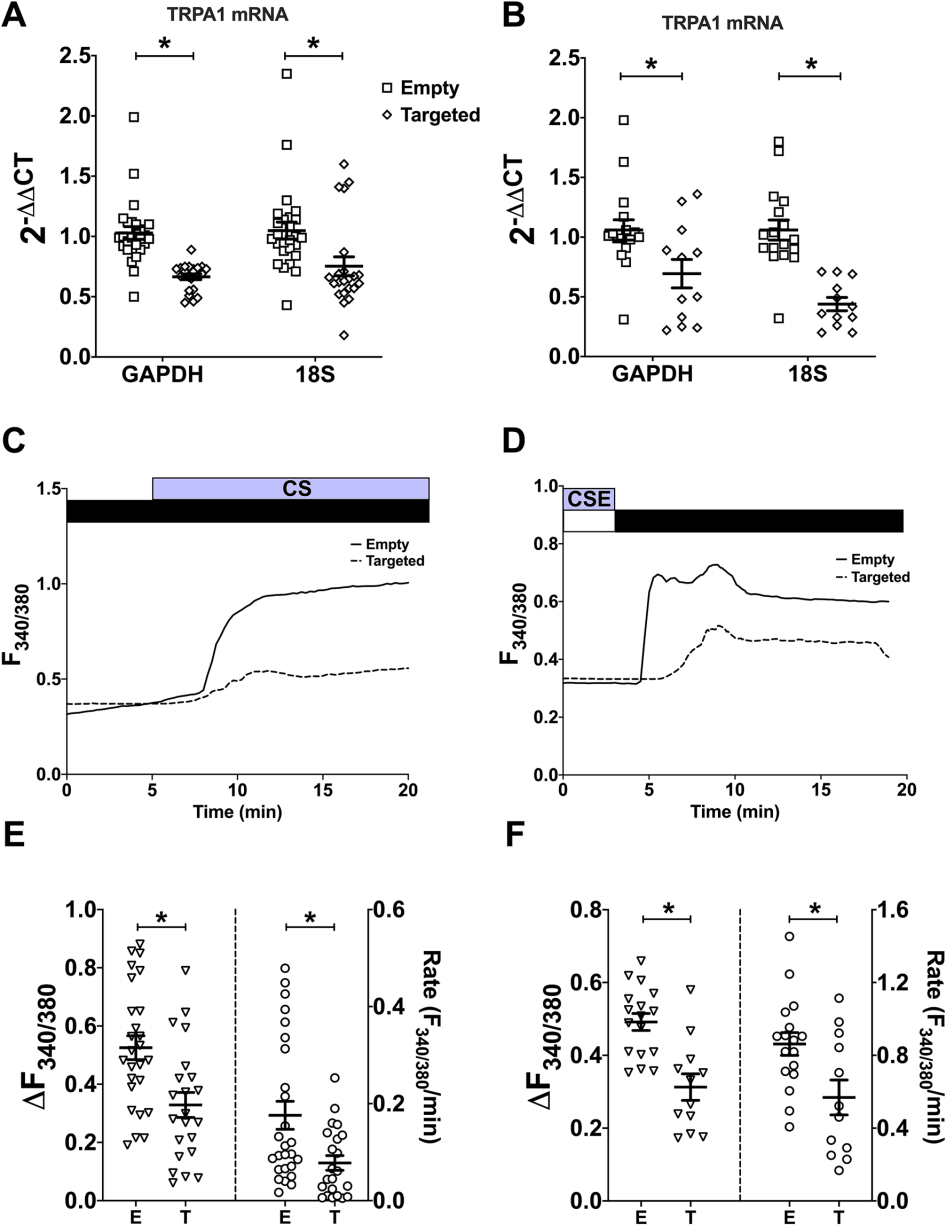
TRPA1 knockdown significantly attenuated CS- and CSE-induced Ca^2+^ influx. hASMC were transfected with shRNA containing either the empty (E) vector, or the vector targeted (T) towards TRPA1. **(A,B)** Knockdown efficiency of TRPA1 in hASMC subjected to CS **(A)** or CSE **(B)** experiments, presented as mean ± SEM. TRPA1 mRNA expression is reported as 2^−ΔΔCT^ (TRPA1 expression relative to the average TRPA1 expression of coverslips transfected with the empty vector in the same plate, day/donor-matched) normalised to two different housekeeping genes, GAPDH and 18S. **(C,D**) Representative Ca^2+^ imaging trace from separate experiments tracking changes in [Ca^2+^]_i_ following puffing one whole cigarette **(C)** or following Ca^2+^ addback after 5-min perfusion with 10% CSE in transfected hASMC **(D)**. **(E,F)** Summary of amplitude and rate of CS- **(E)** or CSE-activated **(F)** Ca^2+^ influx in transfected hASMC, respectively, presented as mean ± SEM. Unpaired t-test was performed between the groups transfected with empty vector and those transfected with the TRPA1 targeted vector (3–4 independent donors; n = 10–26). *p < 0.05.

To further investigate the putative role of TRPA1 in mediating CS- and CSE-induced Ca^2+^ influx, TRPA1 was knocked-down in hASMC through transient transfection of shRNA targeted (T) against TRPA1. A shRNA sequence containing an empty vector (E) was used as a negative control. The relative expression of TRPA1 mRNA, normalised to two housekeeping genes, was significantly lower in cells transfected with the shRNA targeted towards TRPA1 than those transfected with the empty vector (Fig. 6A,B; p < 0.05, E vs. T). In cells where TRPA1 was significantly knocked-down, both the amplitude and rate of CS- (Fig. 6C; 37.4/55.8% inhibition) and CSE- (Fig. 6D; 33.2/34.7% inhibition) induced Ca^2+^ influx were significantly attenuated (Fig. 6E,F; p < 0.05, E vs. T). Collectively, these data suggest that TRPA1 expression is essential for CS/CSE-induced Ca^2+^ influx, corroborating the results from Fig. 5 that TRPA1 is the major contributor to this calcium influx.

### CSE activates myosin light-chain phosphorylation in hASMC

**Figure 7:**
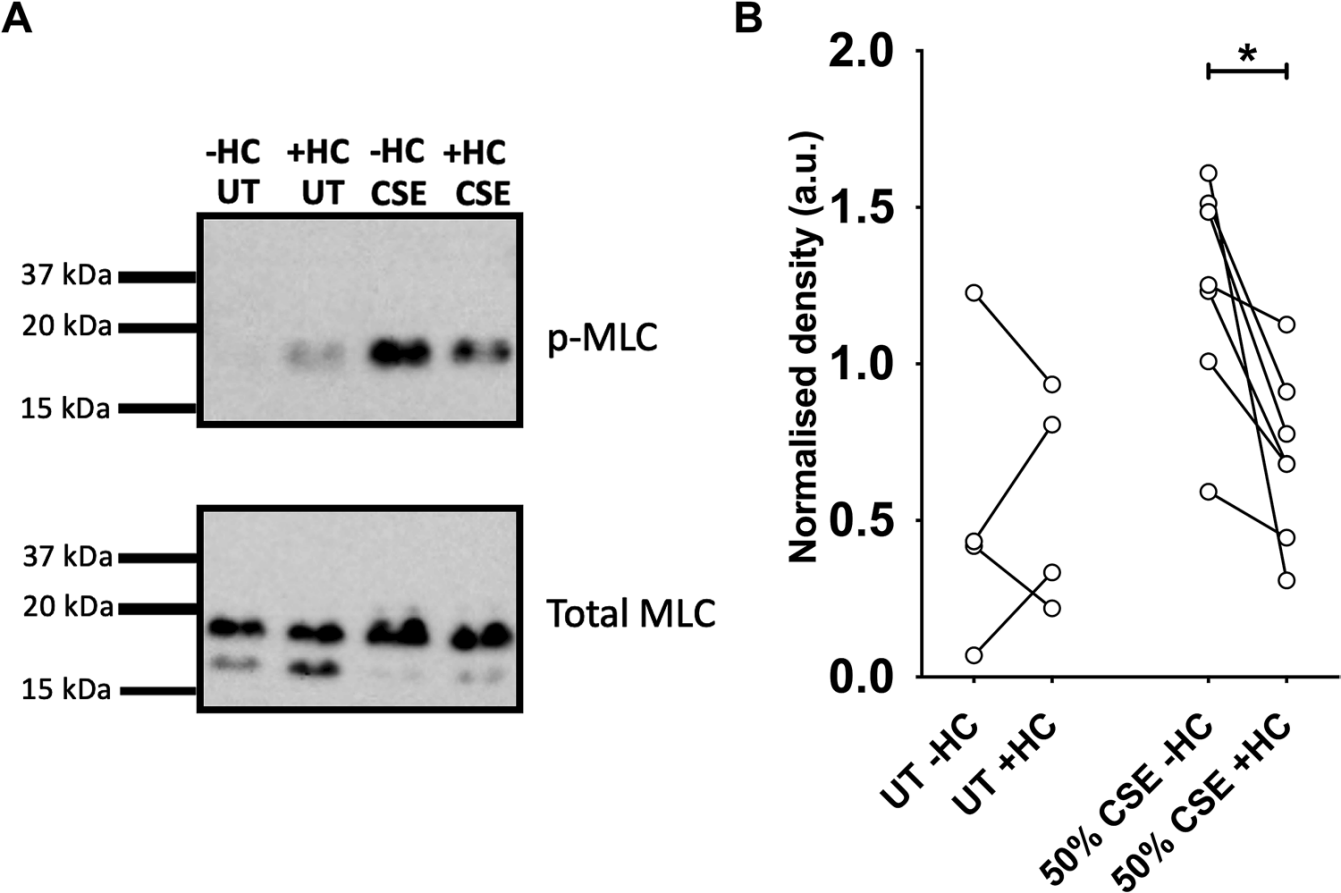
CSE induces MLC phosphorylation in hASMC, downstream of TRPA1-mediated Ca^2+^ influx. Cells were equilibrated with HEPES-buffered solution for 1 h, and lysed after treatment for protein extraction. For the negative control samples without CSE exposure (UT; untreated), cells were treated with 0.1% DMSO (−HC) or 50 µM HC-030031 (+ HC) for 15 min before lysis. For the CSE experiments, cells were pre-treated with 0.1% DMSO (−HC) or 50 µM HC-030031 (+ HC) for 15 min, and then exposed to 50% CSE, with or without HC-030031, for 2 min before lysis. 15 µg of protein was loaded into each well for gel electrophoresis and western blot analysis. **(A)** Representative western blot image for MLC phosphorylation assay described above. All 8 lanes shown were part of the same blot, cut vertically to perform staining with two different antibodies. The 4 lanes in the top panel (p-MLC) match the 4 lanes in the bottom panel (total MLC), i.e. the same protein sample run on the same gel, processed under identical blotting and exposure conditions. Full uncropped blots are presented in Supplementary Fig. 1. **(B)** Semi-quantitative paired comparison of CSE-induced phospho-MLC protein levels, with or without pre-treatment with HC-030031. Band density of p-MLC was normalised to the density of total MLC of the same protein sample (3 independent donors; n = 4–7). *p < 0.05, Wilcoxon matched-pairs signed rank test.

An elevated level of [Ca^2+^]_i_ could bring about adverse downstream effects to the function of ASMC, including the contractile mechanisms of these muscle cells, which is tightly regulated by [Ca^2+^]_i_. An important downstream event subsequent to elevated [Ca^2+^]_i_ is the phosphorylation of MLC. To investigate whether the CSE-induced Ca^2+^ influx was sufficient to induce MLC phosphorylation, a phospho-specific antibody that detects p-MLC was used in western blot analysis of CSE-treated hASMC cell lysate. A 2-min treatment with 50% CSE was applied to capture MLC phosphorylation at peak [Ca^2+^]_i_. Minimal levels of p-MLC protein were detected in lysates of hASMC not treated with CSE, whereas a 50% CSE induced an increase (Fig. 7A). Pre-treatment with the TRPA1 inhibitor HC-030031 reduced the band density of p-MLC, relative to levels of total MLC, in CSE-treated samples, while not affecting non-CSE-treated samples (Fig. 7B). This suggests that CSE-induced Ca^2+^ influx activates downstream phosphorylation of MLC, which could be reduced by inhibiting TRPA1. Altogether, the results provide a mechanistic understanding of CS/CSE-induced disruption of Ca^2+^ homeostasis in primary hASMC, chiefly through TRPA1, which leads to downstream activation of SMC contractile mechanisms and hence, potentially altering the reactivity and tone of ASM.

## Discussion

Our results show that acute exposure to either gaseous CS or to CSE rapidly stimulates Ca^2+^ influx in primary hASMC through activation of TRPA1 channels, which induces downstream phosphorylation of myosin light-chain, presenting TRPA1 as an important link between smoking and airway hyperresponsiveness. TRPA1 is a non-selective cation channel that is primarily associated with noxious cold and mechanical sensation as well as inflammatory pain. Several groups have previously reported that CSE mobilises calcium in neuronal cells, lung epithelia and fibroblasts via TRPA1^23,26–28^, observations which are strongly substantiated by our results. More importantly, we found that exposing primary hASMC to gaseous CS also led to TRPA1-mediated Ca^2+^ influx. To our knowledge, this is the first study that has investigated the acute effect of gaseous CS directly on hASMC Ca^2+^ signalling. In human airway epithelia, gaseous CS produced a relatively slow and sustained increase in [Ca^2+^]_i_^29^, which differs from the rapid influx we observed here in hASMC. The source of this Ca^2+^ response in lung epithelia was suggested to be lysosomal^29^, but our data does not support an intracellular origin of this response in hASMC. In addition to the acute effects, chronic CS exposure was reported to upregulate the expression of TRPA1 in guinea pig tracheal epithelia and in the human epithelial cells^26,30^, as well as TRPC3, CD38, STIM1, and Orai1 in hASMC^16,19^. Therefore, accumulated CS constituents in smokers could also disrupt ASM Ca^2+^ homeostasis in the long-term, in addition to potential acute effects characterised in the present study.

TRPA1 is known to be activated by several components found in CS, namely acrolein, crotonaldehyde, extracellular ROS, and nicotine, in neuronal and lung epithelial cells, and is required to mediate neurogenic inflammation and pain following CS exposure^23,28,31–33^. These constituents, individually or together, are likely responsible for the rapid CS/CSE-induced activation of TRPA1 in hASMC; further exploration studying the role of each individual constituent on hASMC Ca^2+^ influx, as well as modulation of physiological Ca^2+^ responses, is therefore warranted. In particular, acrolein has been implicated in inducing ASM hyper-contractility of the human and rat airway in response to cholinergic agonists, which was attributed partly to augmented Ca^2+^ signalling^34–36^. These components of CS may regulate TRPA1 channel open probability or inhibit channel de-activation mechanisms. For instance, acrolein was found to covalently modify cysteine and lysine residues on TRPA1, which led to sustained membrane currents being activated^33,37,38^. Electrophysiologically, Talavera, et al.^31^ showed that nicotine stabilised the open state and destabilised the closed state of stably expressed mouse and human TRPA1. However, TRPA1 electrophysiology has not been explored in smooth muscle cells. Accordingly, patch-clamping hASMC to study the kinetics, channel activation/deactivation profiles, and Ca^2+^ permeability of endogenous TRPA1 would help elucidate the molecular mechanisms of CS/CSE-induced TRPA1 activation.

HC-030031 has been reported to be highly selective for TRPA1. At 10 µM, the compound did not inhibit other Ca^2+^ channels, including LTCC, NTCC, TRPV1, TRPV3, TRPV4, and TRPM8^39,40^. The calculated IC_50_ in the present study (~ 16 µM) was higher than reported in the initial screening studies (4.9–7.5 µM), albeit in different cell types and against different agonists^39,40^. We also used a genetic approach to ascertain the role of TRPA1, and used mRNA levels to quantify TRPA1 expression; studying TRPA1 protein levels, whenever a validated, specific, antibody emerges, could provide additional evidence for changes in TRPA1 expression in these cells. Our shRNA-driven knockdown of TRPA1 was not fully efficient (mean 2^-ΔΔCT^ for TRPA1-shRNA transfected cells was 0.44–0.75) although we were able to detect a ~ 30–60% attenuation of CS/CSE-induced Ca^2+^ influx with this knockdown model. A complete knockout animal or cell model may provide more definitive evidence of this interaction. CSE and its components were reported to activate TRPA1-mediated Ca^2+^ influx in sensory neurons of WT, but not TRPA1-deficient, mice^23,33^; we hypothesise that ASMC from TRPA1-deficient rodents would not respond to CS/CSE challenge. Moreover, other Ca^2+^ channels may potentially be involved, since we observed that neither HC-030031 nor TRPA1 knockdown completely abolished CS/CSE-induced Ca^2+^ influx. For instance, although our data suggested that SOCC were unlikely to be the major facilitators of acute CS/CSE-induced Ca^2+^ influx, this interpretation assumed that CPA fully activated SOCE in hASMC, which may not be the case. Therefore, SOCC could play a minor role in mediating this influx, but several other candidates to consider include, TRPV1^41^, TRPV4^42^, TRPM8^43^, and reverse-mode NCX^44^, as these proteins were reported to be activated by CSE or CSE constituents in other cell types. Furthermore, as CS-induced Ca^2+^ influx was blocked by Gd^3+^, the involvement of other Gd^3+^-sensitive channels, including stretch-activated cation channels^45^ and various TRPC, TRPM, and TRPV channels^46^, could be further explored.

Direct exposure to gaseous CS and diluted CSE produced comparable Ca^2+^ responses in hASMC (Figs. 1, 2), despite the CS model theoretically delivering higher concentrations of active constituents (1 cigarette in 1 ml buffer), possibly due to saturation of said active constituents. On the other hand, a possible explanation for differences in the time lag between the two delivery systems is that the CS model relies on passive diffusion of water-soluble components into the bathing solution, as opposed to the vigorous bubbling in the CSE model, and therefore it may take some time for the active compounds to equilibrate and reach a threshold concentration for TRPA1 activation. Passive diffusion is more akin to the physiological model, as a number of water-soluble CS constituents can pass through the epithelial barrier in the lungs and diffuse into the circulation, and can be detected in pancreatic juice of smokers^47–49^. These stable circulating active constituents could accumulate in the body, especially for heavy smokers, potentially reaching the activation threshold for Ca^2+^ influx and therefore lead to airway narrowing events.

Since ASM contraction is tightly regulated by [Ca^2+^]_i_, we postulated that CS/CSE-induced Ca^2+^ influx would augment the intrinsic contractility of hASMC, independent of the inflammatory status and signalling from other cell types of the airway. Indeed, we showed that CSE treatment significantly increased the levels of p-MLC, which was reduced by HC-030031, providing an important mechanistic link between acute disruption of Ca^2+^ homeostasis and rapid downstream contractile mechanisms in hASMC. However, whether acute CS/CSE exposure would induce significant airflow limitation requires further research, since our study was performed on tracheal smooth muscle cells, upstream of bronchial/bronchiolar smooth muscle of the smaller airways, which are generally regarded to contribute more prominently to airflow limitation, due to the higher muscle to luminal ratio in the smaller airways. Previously, two groups have reported conflicting results on ASM contraction/relaxation in response to acute CSE exposure^22,23^, although both studies used the intact airway ex vivo, complete with surrounding extracellular matrix, epithelial cells, immune cells, and neurons, all of which could modulate ASM contractility. Indeed, Andre, et al.^23^ previously concluded that the rapid contraction they observed was due to a TRPA1-mediated neurogenic inflammatory response. Physiologically, the ASM functions within an intricate crosstalk network with neighbouring cell types, which may underlie why conflicting results have been published regarding ASM contractility following chronic CSE treatment in organ and cell cultures^12,14,15,21^. The fact that both studies using rat ASM reported hyper-contractility^15,21^, while the studies using bovine ASM and hASMC reported hypo-contractility^12,14^ may indicate species-dependency of CSE-induced AHR.

Due to the prominent expression of TRPA1 in neuronal cells, the potential of TRPA1 modulators in pain therapy has long been suggested^50–52^. In light of emerging evidence, including the present study, for a role of TRPA1 in modulating airway cell function and physiology, both acutely and chronically, the potential for TRPA1 as a therapeutic target for the treatment of respiratory disorders should be considered^53^. Indeed, previous studies from asthma rodent models provide strong support that inhibiting TRPA1 reduces inflammation and bronchoconstriction in response to ovalbumin challenge^54,55^.

In summary, we have identified that TRPA1 underlies the CS/CSE-induced Ca^2+^ response in primary hASMC, which leads to downstream MLC phosphorylation. This elevation of [Ca^2+^]_i_ could potentially lead to exacerbated ASM contraction, especially under inflamed conditions such as those seen in COPD, and hence narrowing of the airway lumen during smoking. The acute, TRPA1-mediated changes in cytosolic Ca^2+^ we have observed may also serve as an initiating factor for further, chronic pathological effects. For instance, elevated [Ca^2+^]_i_ induced by accumulated CS constituents could increase the expression of Ca^2+^ homeostatic regulators in ASMC, in addition to augmenting adverse responses, such as secretion of inflammatory mediators, ROS generation, proliferation, migration, and ECM deposition, all of which contribute to chronic airway hyperresponsiveness and remodelling seen in COPD. Our suggestion that TRPA1 has the potential to be a therapeutic target for treating CS-associated airway diseases, such as COPD, warrants investigation in future studies.

## Methods

### Reagents and antibodies

All reagents were purchased from Sigma-Aldrich (Gillingham, UK) unless otherwise specified. HC-030031, felodipine, and CPA were purchased from Tocris (Abington, UK). The primary antibody against p-MLC (3675S) and the secondary anti-mouse HRP-linked antibody (7076S) were purchased from Cell Signaling Technology (London, UK), while the primary antibody against MLC (M4401) was from Sigma-Aldrich. 3R4F reference cigarettes were purchased from the Kentucky Tobacco Research & Development Center, University of Kentucky. Glycerol stocks of bacteria carrying the plasmid vector encoding shRNA targeted against TRPA1 (TRPA1-shRNA) or the empty control vector (EV) were obtained from Lenti-shRNA Core Facility, UNC Chapel Hill. PCR primers were purchased from Integrated DNA Technologies (Leuven, Belgium).

### Cell culture

Human tracheal rings were procured from non-smoker excess donor lungs from Freeman Hospital, Newcastle upon Tyne and the Cystic Fibrosis Centre, UNC Chapel Hill. Ethical approval was obtained for the collection of non-smoking human donor lung tissues from Newcastle and North Tyneside Local Regional Ethics Committee (16/NE/0230) and the University of North Carolina Institutional Review Board (tissue procurement was done in accordance to protocol 03–139 as described previously^56^). These donors were all adults over the age of 18, and informed consent was obtained for procurement of lung tissues. All experiments presented in this study were performed in accordance with local and institutional guidelines and regulations.

Primary hASMC were isolated from tracheal rings via enzymatic digestion (2 mg/ml collagenase IV, 3 mg/ml elastase, 1 mg/ml trypsin inhibitor, 1 mM EGTA dissolved in M199 media), and cultured in DMEM/F-12 media (supplemented with 100 U/ml penicillin, 100 μg/ml streptomycin, 2 mM L-glutamine, 1% non-essential amino-acid, and 10% fetal bovine serum). Media was replaced every 48–72 h. Cultured primary hASMC were grown in a humidified incubator at 37 °C with 5% CO_2_, and were maintained in culture and used for experiments up to 8 passages. Cultured primary hASMC were stained with α-smooth muscle actin in immunofluorescence up to passage 8 to ascertain smooth muscle identity of the cell population.

For Ca^2+^ imaging experiments and shRNA transfection, hASMC were seeded onto 25 mm glass coverslips, and grown in 6-well plates until ~ 60–90% confluent (Ca^2+^ imaging) or ~ 40–60% confluent (transfection). For the MLC phosphorylation assay, hASMC were seeded directly onto 6-well plates until fully confluent. Cells were serum-starved for 24–48 h before Ca^2+^ imaging experiments or MLC phosphorylation assay.

### Exposure to cigarette smoke (CS)

CS generated from Kentucky 3R4F reference cigarettes was delivered through a Borgwaldt LM1 smoke engine (Hamburg, Germany), into a closed chamber in which hASMC were situated. A glass fibre Cambridge filter pad (0.3 µm pore size) was placed in the tubing to filter out the particulate tar phase. Exposure of hASMC to CS was carried out in 13 puffs with a 30 s interval in between; each puff was 35 ml, delivered over a duration of 2 s. CS persists in the closed chamber after delivery for the full duration of each experiment. Exposure to room air, with identical puff delivery conditions, was used as a control.

### Preparation of cigarette smoke extract (CSE)

The stock solution of 100% CSE was produced by bubbling one 3R4F reference cigarette, with the particulate phase filtered out, into 25 ml of HEPES-buffered solution (in mM: 130 NaCl, 5 KCl, 1 CaCl_2_, 1 MgCl_2_, 10 NaHEPES, 10 D-glucose; for the nominally Ca^2+^-free solution, CaCl_2_ was omitted and replaced with 1 mM EGTA; solutions were adjusted to pH 7.4). The stock solution was made fresh at the beginning of each day of experiments, and kept on ice throughout the day. CSE was diluted into HEPES-buffered solution for use immediately prior to each experiment.

### Ca^2+^ imaging

Primary hASMC grown on glass coverslips were loaded with the cell-permeable [Ca^2+^]_i_ indicator fura-2-AM (5 µM; Thermo Fisher Scientific, Cramlington, UK) for 60 min at 37 °C, washed with HEPES-buffered solution, and left to de-esterify for 15 min at room temperature. Coverslips were mounted onto a Nikon epifluorescence microscope equipped with a 20 × lens and appropriate filters, and the 340/380 emission ratio (F_340/380_) of fura-2 fluorescence was recorded using a Princeton Instruments/Hamamatsu CCD camera. Single cells were circled as areas of interest (15 cells were randomly selected, and were as evenly spaced as possible), and images were collected every 5–15 s (5 s for the influx phase). For CS experiments, cells were left in a static bath (1 ml HEPES-buffered solution) and were covered with a customised chamber with tubing connected to the smoke machine for CS delivery. For CSE experiments, a perfusion system was assembled to allow for fluid exchange (2 ml/min). All experiments were performed at room temperature.

### Isolation of plasmid DNA from bacterial stocks

Glycerol stocks of bacteria carrying plasmids encoding TRPA1-shRNA or empty vector were expanded in LB medium (3 µl glycerol stock in 500 ml LB) overnight on an orbital shaker at 37 °C, supplemented with ampicillin (100 µl/ml) as selection agent. Plasmid DNA was isolated from the harvested bacterial cultures using Plasmid Maxi Kit (Qiagen, Germantown, USA) following manufacturer’s instructions. Precipitated DNA was dissolved in nuclease-free water and stored at − 20 °C until use.

### Transient shRNA transfection

Cultured hASMC were transiently transfected with plasmids encoding TRPA1-shRNA or EV using the Lipofectamine 2000 transfection reagent (Thermo Fisher Scientific) according to manufacturer’s instructions. Plasmid DNA (1.5 µg/well) and Lipofectamine 2000 (3.75 µl/well) were diluted in Opti-MEM (Thermo Fisher Scientific) separately (125 µl for each half/well), mixed together, and then incubated at room temperature for 20 min. The complete culture media DMEM/F-12 on hASMC was replaced with 1 ml DMEM/F-12 without penicillin/streptomycin (DMEM/F-12^-P/S^) per well prior to transfection. The transfection mix was added drop-wise onto each well, swirled, and cells were left overnight in the incubator. DMEM/F-12^-P/S^ was replaced with complete DMEM/F-12 after the transfection period. Cells were studied in Ca^2+^ imaging experiments 72 h post-transfection.

### RNA extraction, cDNA synthesis, and real-time quantitative PCR (qPCR)

Transfected hASMC were lysed after Ca^2+^ imaging experiments, and RNA were extracted using the RNeasy Mini Kit (Qiagen) according to manufacturer’s instructions. Isolated RNA was eluted in nuclease-free water, and stored at − 80 °C until use. 300 ng RNA from each sample was subjected to DNAse (0.5 U/µl; Roche, Welwyn Garden City, UK) treatment to remove DNA contamination (10 min at 37 °C). Reverse transcription was performed by incubating 300 ng of the DNase-treated RNA with a mix of random primers (6.25 µg/µl; Promega, Southampton, UK), RNasin ribonuclease inhibitor (0.5 U/µl; Promega), Deoxynucleotide Triphosphates (300 µM; New England Biolabs, Hitchin, UK) and M-MLV Reverse Transcriptase (5 U/µl; Promega) for 60 min at 37 °C to obtain complementary DNA (cDNA).

Real-time qPCR was performed by mixing the cDNA (1.5 µl), forward and reverse primers (2 µM each), and 2X LightCycler 480 SYBR Green I Master mix (7.5 µl; Roche), to a total volume of 15 µl in each well of a 96-well plate. The housekeeping genes 18S and GAPDH were used as internal controls. Sequences for the primer pairs are listed in Table 1. The PCR was ran using a standard protocol consisting of the activation stage (95 °C for 10 min), followed by 45 cycles of amplification (95 °C for 10 s, 60 °C for 20 s, 72 °C for 1 s) before cooling down. The cycle threshold (CT) values for the detection of each sample was calculated using the built-in Second Derivative Maximum Method in the LightCycler 480 programme (Roche). Relative quantification of TRPA1 in shRNA-transfected samples was performed using the 2^−ΔΔCT^ method^57^, normalising to the two different housekeeping genes (ΔCT), and then normalised to the average TRPA1 ΔCT of samples transfected with the EV in the same plate, day/donor-matched. The Ca^2+^ imaging experiments from TRPA1-shRNA-transfected samples with 2^−ΔΔCT^ > 0.75 to both GAPDH and 18S (i.e. < 25% knockdown in RNA expression) were excluded.

**Table 1:** Sequences of primers used in qPCR analysis. Primer pairs were designed using the NCBI Primer-BLAST tool.

Primer	Sequence (5′ to 3′)	Product length	Position on mRNA/rRNA template
TRPA1 (forward)	GTG GAA CTT CAT ACC AGC TTA GA	99	3113–3135
TRPA1 (reverse)	AGA TCT GGG TTT GTT GGG ATA C		3190–3211
GAPDH (forward)	TGC ACC ACC AAC TGC TTA GC	87	476–495
GAPDH (reverse)	GGC ATG GAC TGT GGT CAT GAG		542–562
18S (forward)	CTC TAG ATA ACC TCG GGC CG	209	293–312
18S (reverse)	GTC GGG AGT GGG TAA TTT GC		482–501

### Western blotting

After treatment, hASMC were lysed using ice-cold RIPA buffer (50 mM Tris, 150 mM NaCl, 1 mM EDTA, 1% Triton-X 100, 0.25% sodium deoxycholate, 0.1% SDS), supplemented with protease and phosphatase inhibitors (Sigma Aldrich). Lysates were centrifuged at 12,000 *g* for 15 min at 4 °C, and pellets were discarded. Protein concentration was quantified using the BCA assay (Pierce BCA Protein Assay Kit, Thermo Fisher Scientific), and 4X Laemmli buffer added. Samples were boiled (95 °C, 5 min), and 15 µg protein was loaded into each well on a 12% SDS-PAGE gel. Gel electrophoresis was run at 150 V for 60 min, and protein was transferred to a PVDF membrane under semi-dry conditions using the Bio-Rad Trans-Blot Turbo Transfer System (25 V constant for 10 min). Membranes were cut vertically to perform staining with two different antibodies (p-MLC and MLC) on the same protein sample run on the same gel. Membranes were blocked with TBS-T with 5% BSA (for p-MLC; 1 h at room temperature) or 5% milk (for MLC; overnight at 4 °C), then incubated with primary antibody diluted in TBS-T (1:500 for p-MLC, overnight at 4 °C; 1:1000 for MLC, 1 h at room temperature). Secondary antibody (1:5000 goat anti-mouse) was incubated for 1 h at room temperature. Enhanced chemiluminescence (ECL) was incubated for 2 min at room temperature, and blots were developed on a Fujifilm LAS-3000 imager. Protein bands were quantified using ImageJ. Band density of p-MLC was normalised to band density of total MLC of the same protein sample run on the same gel.

### Statistical analysis

Summary data is presented as mean ± SEM. All experiments were performed using hASMC isolated from 2 to 4 donors (biological replicates). n denotes number of repeated independent experiments (technical replicates). Statistical analysis was performed using GraphPad Prism 8, with statistical significance indicated by an alpha value of p < 0.05. Non-parametric tests were performed for datasets that did not pass the D’Agostino & Pearson test. The type of analysis for each dataset is indicated in the respective figure legend. Linear regression was performed to estimate the rate of [Ca^2+^]_i_ change.

## Supplementary Information

Supplementary Figure S1.

## Data Availability

Data generated or analysed from this study are available upon reasonable request.

## Abbreviations

COPD: Chronic obstructive pulmonary diseases
ROS: Reactive oxygen species
SOCE: Store-operated calcium entry
[Ca^2^^+^]_i_: Cytosolic Ca^2^^+^ concentration
CPA: Cyclopiazonic acid
MLC: Myosin light-chain
p-MLC: Phospho-myosin light-chain
HRP: Horseradish peroxidase
TRPA1: Transient receptor potential ankyrin 1
EGTA: Ethylene glycol-bis(2-aminoethylether)-*N,N,N′,N*′-tetraacetic acid
DMEM/F-12: Dulbecco’s modified eagle’s medium/Ham’s nutrient mixture F-12
NaHEPES: 4-(2-Hydroxyethyl)piperazine-1-ethanesulfonic acid sodium salt
LB: Lysogeny broth
M-MLV: Moloney-murine leukemia virus
EDTA: Ethylenediaminetetraacetic acid
SDS-PAGE: Sodium dodecyl sulfate–polyacrylamide gel electrophoresis
PVDF: Polyvinylidene difluoride
TBS-T: Tris-buffered saline-Tween20
SR: Sarcoplasmic reticulum
SERCA: Sarco-/endoplasmic reticulum Ca^2^^+^ ATPase
