# Supplementary material for: Acute cigarette smoke or extract exposure rapidly activates TRPA1-mediated calcium influx in primary human airway smooth muscle cells
Source: Sci Rep. 2021 May 5;11:9643. doi: 10.1038/s41598-021-89051-4 (PMC8100124; doi:10.1038/s41598-021-89051-4)
Supplement: Supplementary file 1 — Supplementary Figure S1. [file 41598_2021_89051_MOESM1_ESM.pdf]

Acute cigarette smoke or extract exposure rapidly activates TRPA1-mediated calcium influx in primary human airway smooth muscle cells

JinHeng Lin[1], Michael Taggart[1], Lee Borthwick[1], Andrew Fisher[2], Malcolm Brodlie[2,3], M. Flori Sassano[4], Robert Tarran[4], Michael A. Gray[1*]

[1]Biosciences Institute, Faculty of Medical Sciences, Newcastle University, Newcastle upon Tyne, Tyne and Wear, UK

[2]Translational and Clinical Research Institute, Faculty of Medical Sciences, Newcastle University, Newcastle upon Tyne, Tyne and Wear, UK

[3]Paediatric Respiratory Medicine, Great North Children's Hospital, Newcastle upon Tyne Hospitals NHS Foundation Trust, Newcastle upon Tyne, Tyne and Wear, UK

[4]Department of Cell Biology & Physiology, School of Medicine, University of North Carolina at Chapel Hill, Chapel Hill, North Carolina, USA

***Corresponding author:**

Michael A. Gray

Mailing address: Biosciences Institute, Faculty of Medical Sciences, Newcastle University, Newcastle upon Tyne, NE2 4HH, UK.

Email: m.a.gray@newcastle.ac.uk

Telephone: +44 (0)1912082779

Fax: +44 (0)1912087424

Supplementary Information

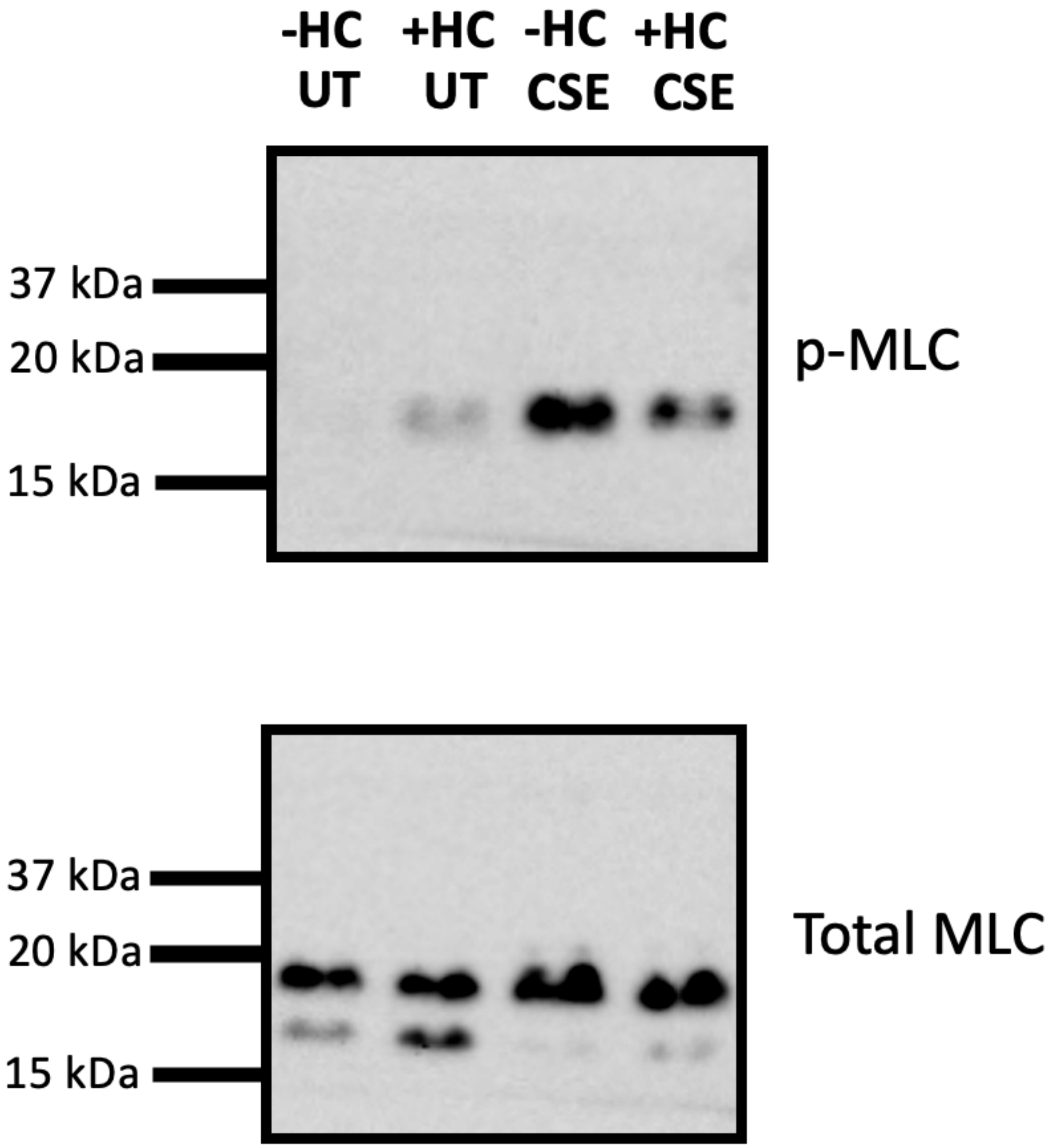

Supplementary Fig. 1 Full uncropped blots for the representative image presented in Fig. 7. All 8 lanes shown were part of the same blot, cut vertically to perform staining with two different antibodies. The 4 lanes in the top panel (p-MLC) match the 4 lanes in the bottom panel (total MLC), i.e. the same protein sample run on the same gel, processed under identical blotting and exposure conditions. Blots were cut just under 15kDa, as under these running conditions, the dye front migrated to the bottom of the gel with 15 and 10kDa still stacked together (Precision Plus Protein WesternC Standards, Bio-Rad).
